# Portable smartphone-based molecular test for rapid detection of *Leishmania* spp.

**DOI:** 10.1007/s15010-024-02179-z

**Published:** 2024-02-14

**Authors:** Rea Maja Kobialka, Arianna Ceruti, Madhurima Roy, Sutopa Roy, Rajashree Chowdhury, Prakash Ghosh, Faria Hossain, Manfred Weidmann, Elena Graf, Jesus Bueno Alvarez, Javier Moreno, Uwe Truyen, Dinesh Mondal, Mitali Chatterjee, Ahmed Abd El Wahed

**Affiliations:** 1 Institute of Animal Hygiene and Veterinary Public Health, Leipzig University, Leipzig, Germany; 2 Institute of Post Graduate Medical Education and Research, Kolkata, India; 3 Nutrition Research Division, International Centre for Diarrheal Disease Research Bangladesh, Dhaka, Bangladesh; 4 Midge Medical GmbH, Berlin, Germany; 5 WHO Collaborating Center for Leishmaniasis, National Center for Microbiology, Instituto de Salud Carlos III, CIBER de Enfermedades Infecciosas-CIBERINFEC, Madrid, Spain

**Keywords:** Isothermal amplification, *Leishmania donovani*, Neglected tropical diseases, Post kala-azar dermal leishmaniasis, Point-of-need test, Visceral leishmaniasis

## Abstract

**Purpose:**

Leishmaniasis, caused by the parasite of the genus *Leishmania*, is a neglected tropical disease which is endemic in more than 60 countries. In South-East Asia, Brazil, and East Africa, it mainly occurs as kala-azar (visceral leishmaniasis, VL), and subsequently as post kala-azar dermal leishmaniasis (PKDL) in a smaller portion of cases. As stated per WHO roadmap, accessibility to accurate diagnostic methods is an essential step to achieve elimination. This study aimed to test the accuracy of a portable minoo device, a small battery-driven, multi-use fluorimeter operating with isothermal technology for molecular diagnosis of VL and PKDL.

**Methods:**

Fluorescence data measured by the device within 20 min are reported back to the mobile application (or app) via Bluetooth and onward via the internet to a backend. This allows anonymous analysis and storage of the test data. The test result is immediately returned to the app displaying it to the user.

**Results:**

The limit of detection was 11.2 genome copies (95% CI) as determined by screening a tenfold dilution range of whole *Leishmania donovani* genomes using isothermal recombinase polymerase amplification (RPA). Pathogens considered for differential diagnosis were tested and no cross-reactivity was observed. For its diagnostic performance, DNA extracted from 170 VL and PKDL cases, comprising peripheral blood samples (VL, *n* = 96) and skin biopsies (PKDL, *n* = 74) from India (*n* = 108) and Bangladesh (*n* = 62), was screened. Clinical sensitivity and specificity were 88% and 91%, respectively.

**Conclusion:**

Minoo devices can offer a convenient, cheaper alternative to other molecular diagnostics. Its easy handling makes it ideal for use in low-resource settings to identify parasite burden.

**Supplementary Information:**

The online version contains supplementary material available at 10.1007/s15010-024-02179-z.

## Introduction

Worldwide, new emerging diseases are a threat to public health systems. Low- and middle-income countries (LMIC) are facing the additional burden of ancient pathogens, circulating in human and animal populations. To control these diseases, the World Health Organization (WHO) created a list of neglected tropical diseases (NTDs) together with a program to eliminate these pathogens targeted for 2030 [1, 2]. One of the NTDs is Leishmaniasis, caused by the protozoan parasite *Leishmania *spp. It is strongly associated with poverty and/or rural settings [3–5]. Transmission between mammals occurs via blood-feeding female phlebotomine sandflies (*Diptera*, *Psychodidae*) [6].

Leishmaniasis has a range of clinical manifestations that include systemic involvement (visceral leishmaniasis, VL), along with various forms of dermal leishmaniasis and mucocutaneous leishmaniasis. In South-East Asia and East Africa, the causative species of VL is *Leishmania donovani* (*L. donovani*), while in the Mediterranean region and the Americas, it is *Leishmania infantum* (also known as *Leishmania chagasi*) [7]. The clinical symptoms for VL include weight loss, intermittent fever, pancytopenia, and hepatosplenomegaly [8]. In India, 2.5–20% of treated VL cases develop a dermal sequela termed as post kala-azar dermal leishmaniasis (PKDL) [9–11]. These patients are epidemiologically important as the parasites are present in the dermis which makes them accessible for ingestion by sandflies as part of the blood meal. Therefore, these patients serve as a reservoir for disease transmission [12–15].

In 2005, WHO published the first global and regional strategies to prevent, control, and eliminate kala-azar in India, Bangladesh, and Nepal as a public health problem. The key strategies toward elimination of VL encompass early diagnosis and complete case management; integrated vector management and vector surveillance; effective disease surveillance through passive and active case detection; social mobilization and building partnerships; and implementation and operational research [16]. The laboratory diagnosis of kala-azar is usually performed by microscopic observation of intracellular amastigotes (Leishman Donovan bodies) in spleen or bone marrow aspirates. Since this method is invasive, serological tests such as the rK39 strip test are preferred as primary diagnostic method [5]. For PKDL, the rK39 strip test falters in terms of specificity, as positivity may be attributable to a previous VL infection with presence of circulating anti-leishmanial antibodies [17]. Therefore, PKDL cases are diagnosed empirically based on clinical suspicion followed by detection of *L. donovani* parasites in slit skin smears or skin biopsies [18].

Clinical presentations for PKDL include any combination of macules, papules and/or nodules termed polymorphic PKDL, or hypopigmented lesions termed macular PKDL. Even though the parasite load is significantly lower in macular lesions [19], both manifestations comprise an equal proportion of the disease burden, are infectious to sandflies [14, 20], and therefore contribute to disease transmission [13]. Diagnosis of PKDL and monitoring of treatment are challenging, attributable to patients having poor health seeking behavior as the disease is not life-threatening [20, 21]. Furthermore, the low parasite load in macular PKDL cases leads to false negativity in slit skin smears, as the amastigotes are difficult to observe microscopically [22, 23]. In addition, these macular PKDL lesions can closely mimic leprosy, vitiligo, pityriasis versicolor, and psoriasis, thereby presenting a diagnostic dilemma [24].

To ensure the success of the kala-azar elimination program in South-East Asia (KAEP), there is a need for an accurate, rapid, and practical detection method to diagnose VL and PKDL [25]. In terms of compliance of the patient to treatment, the time to result delivery plays an important role. In rural areas, medical care is not sufficient, and it takes time and financial support to get to a medical facility. As PKDL occurs in the marginalized sections of society, there is a need for rapid detection methods that could shorten the turnaround time from diagnosis to treatment [26].

Real-time qPCR is a highly sensitive and specific detection method for parasite DNA [27, 28], but usually requires trained personnel and large footprint laboratory equipment that is very challenging to be implemented in resource limited settings. Isothermal amplification techniques, e.g., Recombinase Polymerase Amplification (RPA) amplify DNA at a constant temperature with the use of much simpler equipment than qPCR. RPA is highly specific and sensitive making it a valuable tool for molecular detection in low-resource settings [29]. The devices typically used for RPA are portable and relatively small, but still limited to use in small medical facilities or in a mobile laboratory.

In this study, we assessed the diagnostic potential of the rapid, smartphone-based minoo device for human VL and PKDL using isothermal RPA, which can be utilized as a point-of-need test. The minoo is a small, battery-driven fluorometer with a thermal control system, measuring fluorescence signal during isothermal amplification. The sensitivity and specificity of minoo were determined under laboratory conditions and then with archived clinical samples.

## Materials and methods

### Ethical approval

The study was approved by the Institutional Ethics Committee of Institute of Post Graduate Medical Education and Research IPGMER, Kolkata, India (Approval ID: IPGME&R/IEC/2021/273) and the institutional review board (IRB) committee of International Centre for Diarrheal Disease Research, Bangladesh (icddr,b), Dhaka, Bangladesh (No: PR-14093). All study participants or their legally accepted guardian provided written informed consent.

### Study population

In India, archived samples previously used in an RPA-based study [30] were tested by the minoo device at IPGMER, Kolkata, India. In total, *n* = 36 extracted samples from whole blood and *n* = 60 from skin biopsies were included. The samples from Bangladesh were archived samples at the icddr,b, Dhaka, Bangladesh. Here DNA extracts from whole blood, *n* = 72 [31] and skin-biopsy samples, *n* = 2 [32] were included.

### Spin column-based DNA extraction method

In India, the QIAmp DNA mini kit (Qiagen, Hilden, Germany) was used for DNA extraction from 200 µL heparinized blood of patients suspected/diagnosed with VL and from skin biopsies in phosphate-buffered saline (20 mM, pH 7.4, PBS) of PKDL suspected cases according to manufacturer’s instructions. After excising skin biopsies into small pieces, DNA was eluted in 50 µL of DNA elution buffer.

In Bangladesh, DNA was isolated from 200 μL of whole blood samples using the QIAamp DNA tissue and blood mini kit. DNA from skin samples was extracted using DNeasy Blood and Tissue Kits (Qiagen, Hilden, Germany) following the manufacturer’s instructions, with a minor modification: skin-biopsy materials were incubated at 37 °C overnight after addition of lysis buffer (ATL) and proteinase K. On the following day, the material was homogenized and incubated at 56 °C for 2 h before purification according to the manufacturer’s instructions. DNA was eluted in 200 µL PCR grade water or elution buffer. Extracted DNA samples were stored at − 20 °C until downstream analysis.

### Real-time PCR

In India, real-time qPCR was undertaken using the ABI StepOnePlus Real-Time Thermal Cycler (Thermo Fisher Scientific, Waltham, USA) and performed using forward primer: 5′-CTTTTCTggTCCTCCgggTAgg-3′, reverse primer: 5′-CCACCCggCCCTATTTTACACCAA-3′ and TaqMan probe (5′-6FAM-TTTTCgCAgAACgCCCCTACCCgC—BBQ-3′) with the LightCycler Multiplex DNA Master (Roche, Basel, Switzerland). In a total volume of 20 µL, 15 µL of the reaction mixture (TaqMan Master Mix, containing 100 nM of each primer and 50 nM of probe) and 5 µL of DNA was used. The protocol included a heating step of 95 °C for 1 min, 45 cycles of a denaturation step at 95 °C for 10 s, and an annealing and extension step of 60 °C for 45 s. The last step was a cooling step at 40 °C for 30 s. The cutoff value was set at a cycle threshold (Ct) value > 30.

In Bangladesh, targeting conserved region of *Leishmania* RLEP repeats (L42486.1) specific for *L. donovani* and *L. infantum,* Taqman primers and probes were designed to perform the real-time PCR following a method described by Vallur et al. [33]. Briefly, a 20 μL reaction mix was prepared containing 9 μL template, 10 μL of TaqMan^®^ Gene Expression Master Mix (2×), 1 μL pre-ordered primer–probe mix and PCR grade water. Amplification was performed on a Biorad CFX96 iCycler system with following reaction conditions: 10 min at 95 °C, followed by 45 cycles of 15 s at 95 °C and 1 min at 60 °C. The total reaction time for real-time PCR was approximately 120 min. Specimens ran as duplicates. To quantify the parasite load, each run included one standard curve with DNA concentration corresponding to parasite load 10,000 to 0.1 parasites per reaction. Each run also included one reaction with molecular grade water as a negative control. Samples with cycle threshold (Ct) > 40 were considered as negative.

### Isothermal DNA amplification

The method of detection in minoo devices was fluorescent signal measurement after isothermal DNA amplification. The TwistAmp Exo kit (TwistDx Ltd., Cambridge, UK) and *Leishmania*—RPA oligonucleotides (TibMolBiol, Berlin, Germany) were used as previously reported [34]. A total reaction volume of 50 µL was prepared using the TWIST Amp exo kit (Maidenhead, UK) that included rehydration buffer (29.5 µL), magnesium acetate (2.5 µL, final concentration 14 mM), 10 µM forward primer (2.1 µL), 10 µM reverse primer (2.1 µL), 10 µM probe (0.6 µL) (final concentrations primers 420 nM, probe 120 nM), and molecular nuclease free water (10.7 µL). All reagents were added into the lid of the reaction tube containing freeze-dried reaction pellets. The tube was closed, centrifuged, vortexed, and centrifuged again, then the content was transferred to a 200 µL PCR tube. Thereafter, 2.5 µL of template was added and mixed by pipetting. The tube was then incubated in the minoo device (midge medical GmbH, Berlin, Germany) for amplification at a constant temperature of 42 °C for 18 min.

### Smartphone-based analysis

The minoo device is a portable diagnostic test station that facilitates the detection of biological reactants in a simplified and robust manner. The onboard battery is chargeable via USB port, one charge being sufficient for six independent runs, thus providing independence from unstable power supply. It includes an optical unit and a thermal control system to conduct and monitor isothermal amplification reactions in standard 200 µL PCR tubes. The thermal system incorporates a coil and onboard temperature sensors, which are strategically positioned near the sample for precise temperature control. The optical system of the device is based on a Light Emitting Diode (LED) and an onboard multichannel digital spectrometer.

**Fig. 1:**
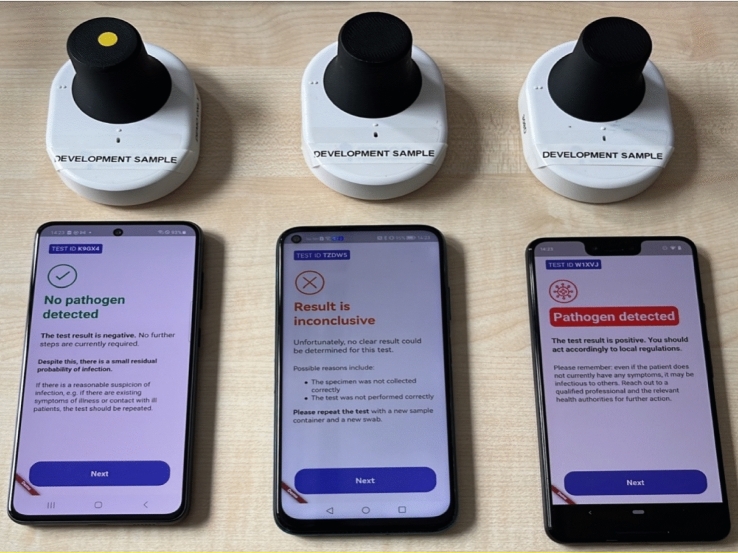
Minoo devices in use with three result options displayed in the application

The minoo test station is wirelessly (Bluetooth) controlled from an authorized application, which communicates the relevant data to the backend via the internet (Wi-Fi) for analysis and usage monitoring. Kassandra, a proprietary Python-based algorithm, is utilized to analyze the fluorescence amplification signal. Kassandra performs three main steps: data processing, where the background from the primary channel is removed and quality checks are performed; feature extraction, where relevant feature values are extracted from the amplification curve to differentiate positive and negative results; and classification, where a test result is calculated based on empirically defined thresholds and weights. After analysis, the result of the test (positive, negative, or inconclusive) is directly displayed on the smartphone application (See Fig. 1).

### Molecular standards and limit of detection (LOD)

Whole genome DNA from a *Leishmania donovani* strain MHOM/IN/80/DD8 (WHO collaborating centre for leishmaniasis, Laboratorio de Referencia e Investigación en Parasitología Centro Nacional de Microbiología, Instituto De Salud Carlos III, Spain) and kinetoplast DNA standard (kDNA) (GeneArt, Regensburg, Germany) were used to determine the sensitivity of the assay (supplement 1). Tenfold serial dilutions ranging from 10^7^ to 10^0^ DNA molecules/µL were prepared and used to determine the assay’s limit of detection.

### Pathogens for detection of cross-reactivity and analytical specificity

To determine the analytical specificity, nucleic acids of 12 pathogens, including different *Leishmania* species, were tested with the minoo assay (Table 1). All *Leishmania* species and *Trypanosoma cruzi* were provided by Laboratorio de Referencia en Leishmaniasis WHO Collaboration Centre for Leishmaniasis, Centro Nacional de Microbiología, Instituto De Salud Carlos III, Spain. *Mycobacterium tuberculosis* and *Mycobacterium leprae* were provided by Quality Control for Molecular Diagnostics (QCMD), Glasgow, United Kingdom and IPGMER, Kolkata, India, respectively. *Plasmodium malariae* and Monkeypox virus were contributed by the Robert Koch Institute, Berlin, Germany, and the German Primate Center, Göttingen, Germany, respectively.

**Table 1:** Pathogens tested for cross-detection in the minoo assay

Pathogen	Result minoo
*Leishmania donovani*^a^	Pos
*Leishmania tropica*^a^	Pos
*Leishmania major*^a^	Pos
*Leishmania aethiopica*^a^	Pos
*Leishmania infantum*^a^	Pos
*Leishmania brasiliensis*^a^	Pos
*Leishmania amazonensis*^a^	Pos
*Trypanosoma cruzi*^a^	Neg
*Mycobacterium tuberculosis*^b^	Neg
*Mycobacterium leprae*^c^	Neg
*Plasmodium malariae*^d^	Neg
Monkeypox virus^e^	Neg

^a^Provided by Laboratorio de Referencia en Leishmaniasis WHO Collaboration Centre for Leishmaniasis. Centro Nacional de Microbiología, Instituto De Salud Carlos III, Spain

^b^Provided by Quality Control for Molecular Diagnostics (QCMD), Glasgow, United Kingdom

^c^Provided by IPGMER, Kolkata, India

^d^Provided by the Robert Koch Institute, Berlin, Germany

^e^Provided by the German Primate Center, Göttingen, Germany

### Statistical analysis

To determine the sensitivity, specificity, positive predictive value (PPV), and negative predictive value (NPV), standard formulas were used [35]. RStudio version 1.3.1093 (RStudio, Boston, MA, United States) (64) was used to perform probit regression and calculation of the LOD. Data were visualized using the ggplot2 package (v3.3.3; [36]).

## Results

### Diagnostic performance of *Leishmania*-RPA on the minoo device

#### Analytical sensitivity and specificity

The LOD was determined using a tenfold dilution of a whole genome *L. donovani* standard and the synthetic molecular kDNA (kinetoplast DNA) standard. The LOD was 11.2 copies/reaction (95% CI) of the whole genome (Fig. 1a) and 134 copies/reaction (95% CI) of the kDNA standard (Fig. 1b). The reaction time was 18 min, with a preparation time of approximately 5 min per sample.

**Fig. 2:**
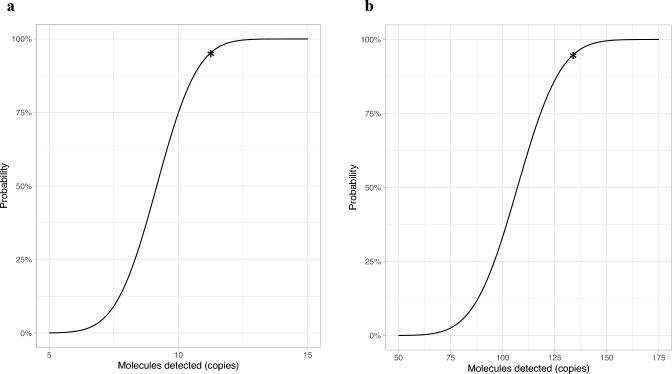
Probit analysis based on the results of four runs with a dilution range of the whole genome from 10^3^ to 10^0^ copies/reaction calculated a LOD (at 95% probability) of 11.2 copies/reaction (**a**). Probit analysis based on the results of three runs with a dilution range of kDNA standard from 10^4^ to 10^1^ copies/reaction with a LOD (95%) of 134 copies/reaction (**b**). *LOD (95%)

All tested *Leishmania* species were detected. No cross-reactivity was observed with nucleic acids from other pathogens (Table 1; Fig. 2).

#### Clinical samples

**Fig. 3:**
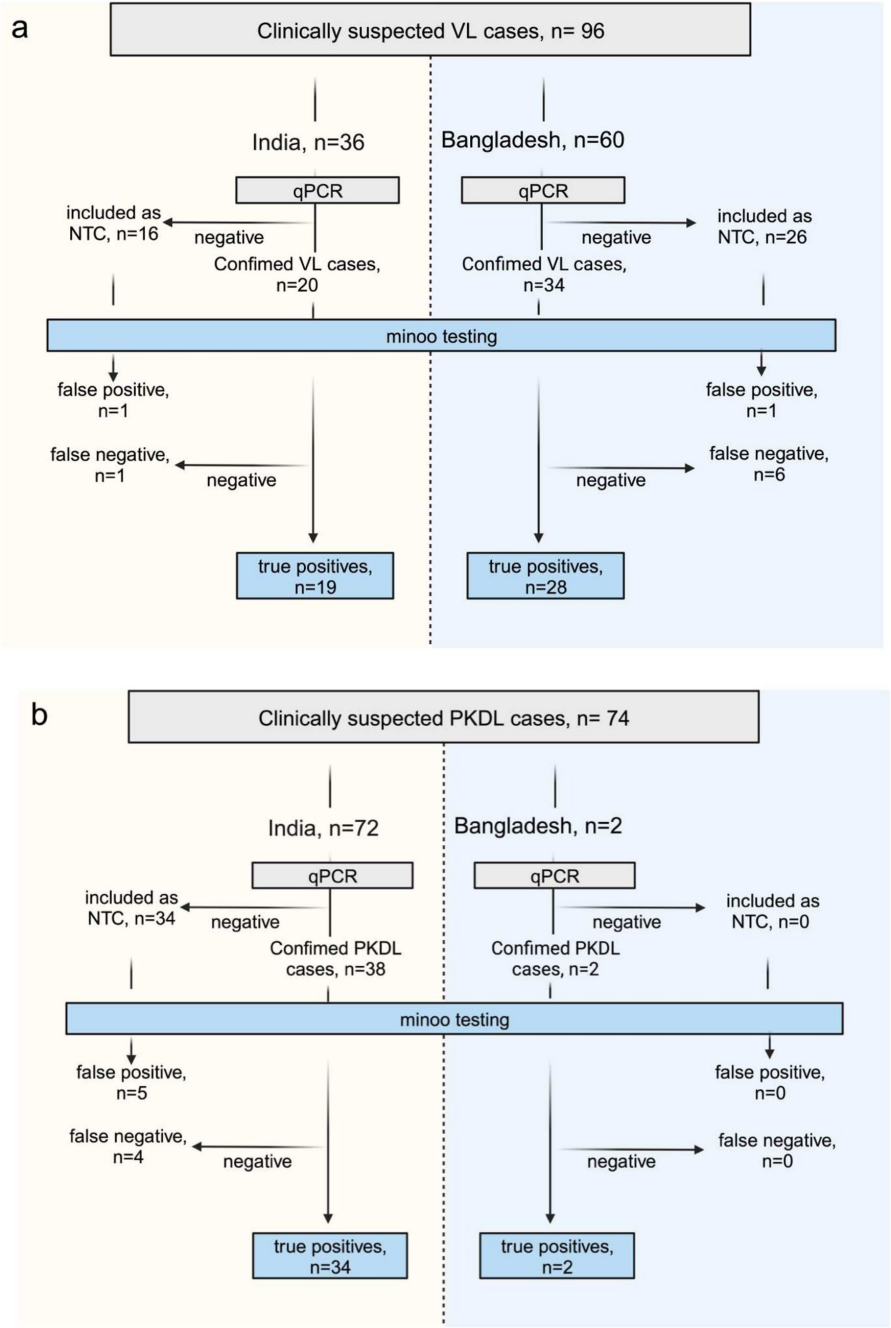
Schematic flowchart of clinical samples which were tested by qPCR and minoo in India and Bangladesh. It is divided into samples from cases with visceral leishmaniasis, VL (**a**) and post kala-azar dermal leishmaniasis, PKDL (**b**). *n* number of samples, *qPCR* quantitative polymerase chain reaction

**Fig. 4:**
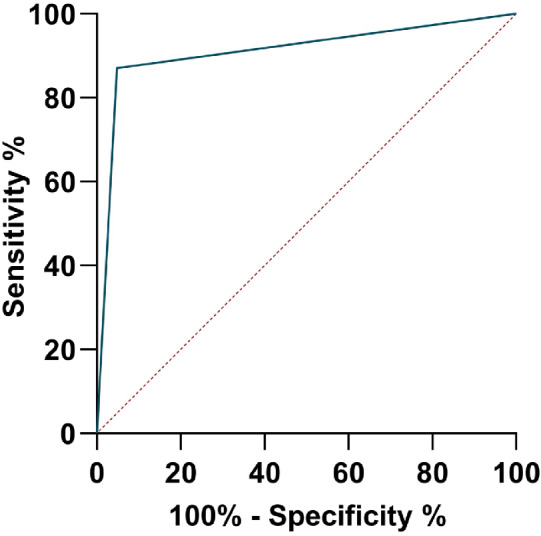
Receiver operating characteristic curve (ROC) for minoo for detection of *Leishmania* DNA in samples from cases with visceral leishmaniasis from India and Bangladesh

**Fig. 5:**
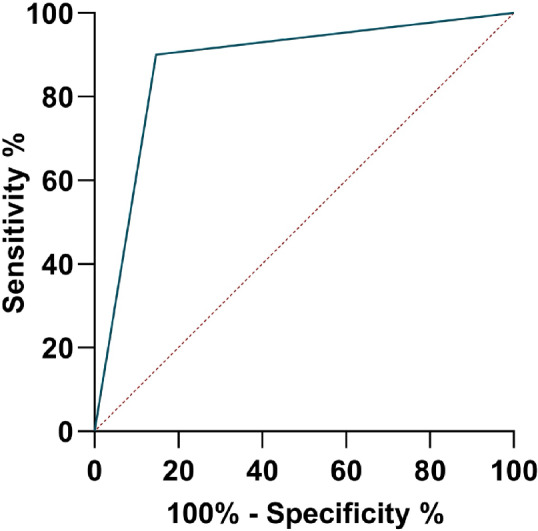
Receiver operating characteristic curve (ROC) for minoo for detection of *Leishmania* DNA in samples from cases with post kala-azar dermal leishmaniasis from India and Bangladesh

In total, 170 human samples (blood: *n* = 96; skin biopsies: *n* = 74) were screened by minoo in India and Bangladesh. Of these samples, 94/170 were positive by real-time qPCR and 83/94 were detected positive by minoo, 76/170 were detected negative by qPCR and 69/76 were detected negative by minoo (Fig. 3). This yields to an overall sensitivity and specificity of the RPA on the minoo across all samples of 88% and 91%, respectively (Table 2). The area under the receiver operating characteristic curve (AUC value) for VL was found to be 0.91 (95% CI 0.8466–0.9761) and for PKDL, it was 0.87 (95% CI 0.7885–0.9645) (Figs. 4 and 5). An inverse relationship between the Ct value in qPCR and the sensitivity of minoo in detecting *Leishmania* DNA was found (Table 3).

**Table 2:** Clinical sensitivity, specificity, positive predictive value (PPV), and negative predictive value (NPV) of minoo

	VL and PKDL			VL			PKDL		
	IN and BD (*n* = 170)	IN (*n* = 108)	BD (*n* = 62)	IN and BD (*n* = 96)	IN (*n* = 36)	BD (*n* = 60)	IN and BD (*n* = 74)	IN (*n* = 72)	BD (*n* = 2)
Number of positive samples qPCR	94	58	36	54	20	34	40	38	2
Number of positive samples minoo	83	53	30	47	19	28	36	34	2
Sensitivity minoo	88%	91%	83%	87%	95%	82%	90%	89%	100%
Specificity minoo	91%	88%	96%	95%	94%	96%	85%	85%	None
PPV	0.92	0.89	0.96	0.96	0.95	0.96	0.88	0.87	1
NPV	0.86	0.89	0.80	0.85	0.93	0.80	0.88	0.88	None

**Table 3:** Comparison of Ct values of real-time qPCR and sensitivity of minoo

	Number of samples	Ct	False negatives	Sensitivity (%)
IN	20	0–20	1	94.7
	22	20–25	2	90.9
	16	25–30	2	87.5
BD	7	25–30	1	85.7
	29	30–40	5	82.7

## Discussion

The WHO Elimination Program for NTDs is gradually approaching their goal since the development of the first NTD roadmap for prevention and control [2]. However, more than 1 billion people worldwide still suffer from the burden of NTDs. Accurate, simple, and cost-effective diagnostic tools are essential to reach and maintain the goals of prevention, control, and elimination of these pathogens. In the roadmap for Neglected Tropical Diseases 2021–2030, the WHO emphasizes the need for innovative molecular diagnostic approaches to achieve these goals [2]. A WHO-working group—the Diagnostic Technical Advisory Group for Neglected Tropical Diseases (DTAG) is responsible for identifying and emphasizing diagnostic needs. In India, NVBDCP and the Kala-azar Task Force are involved in these efforts. For countries like Nepal and Bangladesh, which have already achieved elimination status, it is crucial to continue their efforts of surveillance and prevention [37].

In this study, we evaluated the efficacy of a portable device that enables molecular point-of-need diagnostics. This is highly relevant for monitoring of VL cases as there is no rapid antigen-based test available for the detection of the parasites in peripheral blood. Serological antibody tests (like the rK39 strip test) falter in terms of sensitivity—especially in patients with immunodeficiency or HIV infections [38]. In addition, serological assays do not distinguish between active and past infections, which might lead to false positive results [39–41]. Moreover, there is a problem of cross-reactivity with antibodies from other parasitic infections like Chagas and mycobacterial infections like Leprosy or tuberculosis [42–44]. The analytical sensitivity of the *Leishmania*-RPA evaluated on the minoo device (11.2 genome copies/reaction) suggests its suitability for the detection of parasites in PKDL lesions, as the mean parasite load in skin biopsies is approximately 50 per µg genomic DNA, with higher load in polymorphic cases and a lower load in macular cases [32].

For VL patients, the parasite load in the blood varies widely between 39 parasites/mL and 2.16 × 10^5^ parasites/mL [45]. The clinical sensitivity and specificity of the minoo assay were 88% and 91%, respectively. In regard to the false negative results, several aspects can be considered. As there is a relation between Ct values of real-time qPCR and false negative samples in minoo, low parasite load (especially in VL samples) could be the cause. However, as the number of samples tested varies between the different Ct ranges, they cannot be directly compared. Another impact is the storage time of the extracted samples. Especially in Bangladesh, high Ct values of the samples were observed. All of them were archived samples that had previously been used for other studies, stored at − 20 °C. It is known that the process of freezing and thawing influences the integrity of nucleic acids which could then impact the amplification profiles [46–48]. Therefore, it is likely that the use of fresh samples would significantly improve the sensitivity [49].

While molecular testing usually is confined to a well-equipped laboratory, recent advancements make the development of portable tests possible [50]. For the diagnosis of VL, Recombinase Polymerase Amplification (RPA) has proven to be a reliable diagnostic method [30, 34]. However, these assays used a more expensive device that, although portable, was still tied to the use of a suitcase laboratory. The major advantage of the minoo device is that it has been designed with usability at the core of the design which in principle allows on-site testing by a relatively inexperienced lay user. Data transmission to the backend did not suffer from internet connection instabilities experienced during testing and all data sets were recorded in full on the backend. Lyophilized RPA reagents are cold-chain independent, and oligonucleotide containing lyopellets additionally simplifies the protocol [51]. With this, the minoo assay addresses seven of the REASSURED criteria for diagnostics (real-time connectivity, affordable, sensitive, specific, user-friendly, rapid and robust, and deliverable to end users), but in the case of leishmaniasis, testing does not yet cover ease of specimen collection and the category “equipment-free” [52]. However, the rechargeable device is an alternative to platforms which are nominally described as equipment-free, but actually use a one-use only disposable device [53].

Furthermore, different features (e.g., temperature) of the minoo devices can be easily adapted, allowing the use of other RPA assays for different pathogens. This is especially important in areas with a high prevalence of multiple diseases. Here, it is essential to focus on widespread diagnostic tools instead of disease-specific diagnostics [2].

Another advantage of this platform is the digital/cloud-based interface, which could enable transmission of results to Ministry of Health (MOH) surveillance programs and facilitate data processing. Comprehensive databases are crucial for disease risk assessment and evaluation of containment measures [54]. Furthermore, NTDs are considered as tracers to reveal general healthcare shortages. Thus, a high prevalence of an NTD highlights the need for healthcare improvement [2]. Although the results are highly promising, the assay has some limitations. One critical point is the handling of sensitive data. To address this point, the Smartphone App generates an anonymous ID code for each test, and personal data are not handled on the backend, thus providing personal data protection. Possible transmission of data to other servers in the future would need to be set up according to local data protection regulations. Currently 95% of the world population have access to broadband (3G) mobile phone network coverage although some blind spots remain [55]. Therefore, in most cases, internet access through the Smartphone App should be possible.

For detection of *Leishmania*, blood or skin samples are the target samples. These invasive procedures limit *Leishmania* diagnostics to clinical facilities. However, VL can be detected from whole blood which might allow the use of capillary drop diagnostics (ongoing work). This would enable testing in a home-based setting. However, DNA extraction is the bottleneck of a simplified testing approach and needs to be addressed. The use of a rapid extraction method which has been used by previous studies for the extraction of DNA in tissue samples is a promising method to address this issue [56, 57].

## Conclusion

The *Leishmania*-RPA on the minoo device has the potential to contribute to the progress of controlling the spread of *Leishmania donovani* and achieving/maintaining elimination status through its routine use in diagnosis and treatment monitoring for VL and PKDL. The smartphone-based result readout enables good applicability for use at the point-of-need. However, further studies need to be performed to confirm results and to address the bottleneck of sample collection and extraction. In addition, a multi-country clinical study is warranted prior to its broader point-of-need implementation in endemic settings.

### Supplementary Information

Below is the link to the electronic supplementary material.

Supplementary file1 (PDF 140 KB)

Supplementary file2 (PDF 416 KB)

## Data Availability

No datasets were generated or analysed during the current study.
