# Supplementary material for: Portable smartphone-based molecular test for rapid detection of Leishmania spp
Source: Infection. 2024 Feb 14;52(4):1315–24. doi: 10.1007/s15010-024-02179-z (PMC11288998; doi:10.1007/s15010-024-02179-z)
Supplement: Supplementary file 2 — Supplementary file2 (PDF 416 KB) [file 15010_2024_2179_MOESM2_ESM.pdf]

Research article

Portable smartphone-based molecular test for rapid detection of *Leishmania* spp.

Rea Maja Kobialka[1]*, Arianna Ceruti[1], Madhurima Roy[2], Sutopa Roy[2], Rajashree Chowdhury[3], Prakash Ghosh[3], Faria Hossain[3], Manfred Weidmann[4], Elena Graf[4], Jesus Bueno Alvarez[4], Javier Moreno[5], Uwe Truyen[1], Dinesh Mondal[3], Mitali Chatterjee[2], Ahmed Abd El Wahed[1]

[1] Institute of Animal Hygiene and Veterinary Public Health, Leipzig University, Germany

[2] Institute of Post Graduate Medical Education and Research, Kolkata, India

[3] Nutrition Research Division, International Centre for Diarrheal Disease Research Bangladesh, Dhaka, Bangladesh

[4] midge medical GmbH, Berlin, Germany

[5] WHO Collaborating Center for Leishmaniasis, National Center for Microbiology, Instituto De Salud Carlos III, Spain CIBER de Enfermedades Infecciosas – CIBERINFEC

*Correspondence: rea_maja.kobialka@vetmed.uni-leipzig.de

Supplementary 2

a

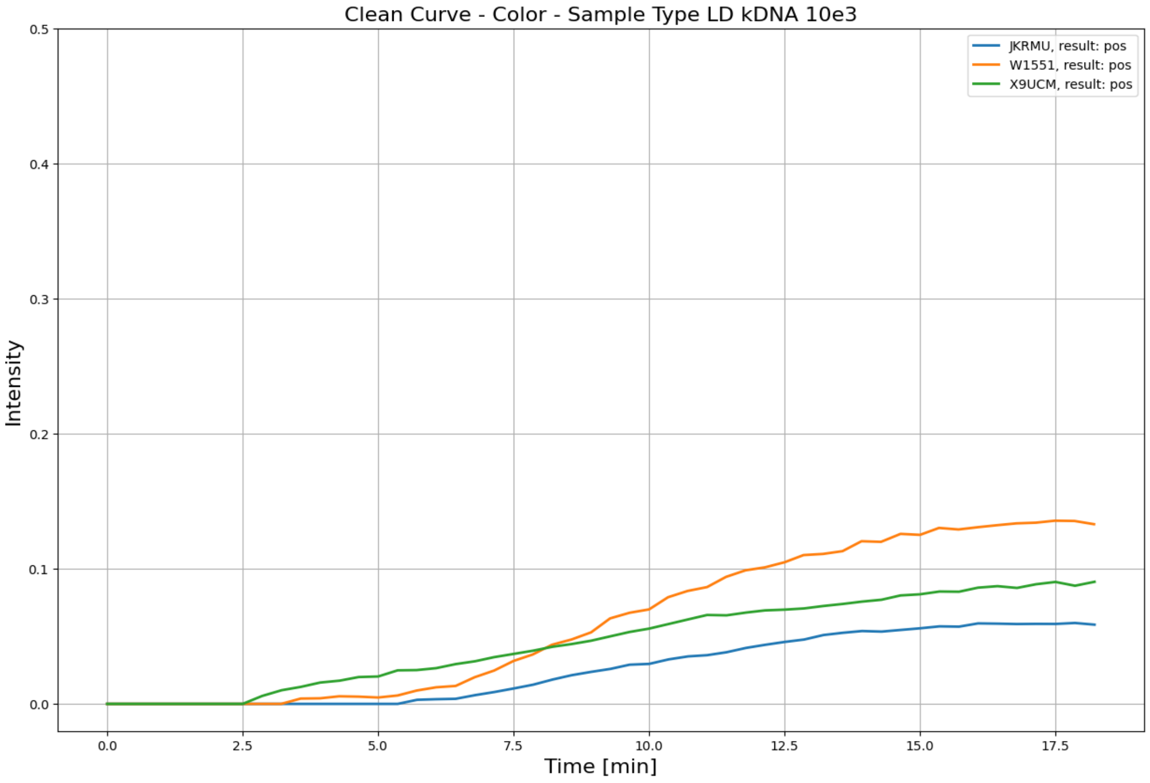

b

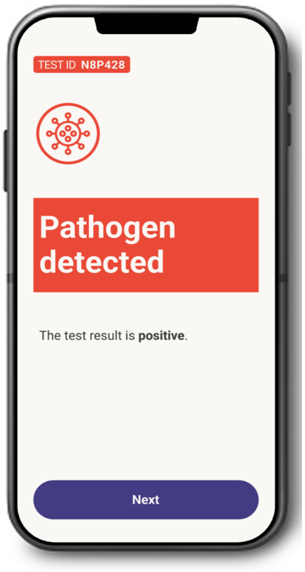

Fig 2 Performance of three minoo devices with 10^3 copies/µL of kDNA standard. The curves show the intensity of fluorescent signal after analysis of Kassandra in the backend (**a**). User interface where the result is shown directly on the app (**b**).
